# Dynamics for High-Sensitivity Detection of Free Radicals in Primary Bronchial Epithelial Cells upon Stimulation with Cigarette Smoke Extract

**DOI:** 10.1021/acs.nanolett.4c02409

**Published:** 2024-07-16

**Authors:** Y. Zhang, A. Sigaeva, S. Fan, N. Norouzi, X. Zheng, I. H. Heijink, D. J. Slebos, S. D. Pouwels, R. Schirhagl

**Affiliations:** † Department of Biomaterials and Biotechnology, University of Groningen, University Medical Center Groningen, Antonius Deusinglaan 1, 9713AV Groningen, The Netherlands; ‡ Department of Pathology and Medical Biology, University of Groningen, University Medical Center Groningen, Hanzeplein 1, 9713GZ Groningen, The Netherlands; § Department of Pulmonology, University of Groningen, University Medical Center Groningen, Hanzeplein 1, 9713GZ Groningen, The Netherlands; ∥ Groningen Research Institute for Asthma and COPD (GRIAC), University of Groningen, University Medical Center Groningen, Hanzeplein 1, 9713GZ Groningen, The Netherlands

**Keywords:** Diamonds, NV centers, Nanodiamonds, Lung cells, COPD, Relaxometry

## Abstract

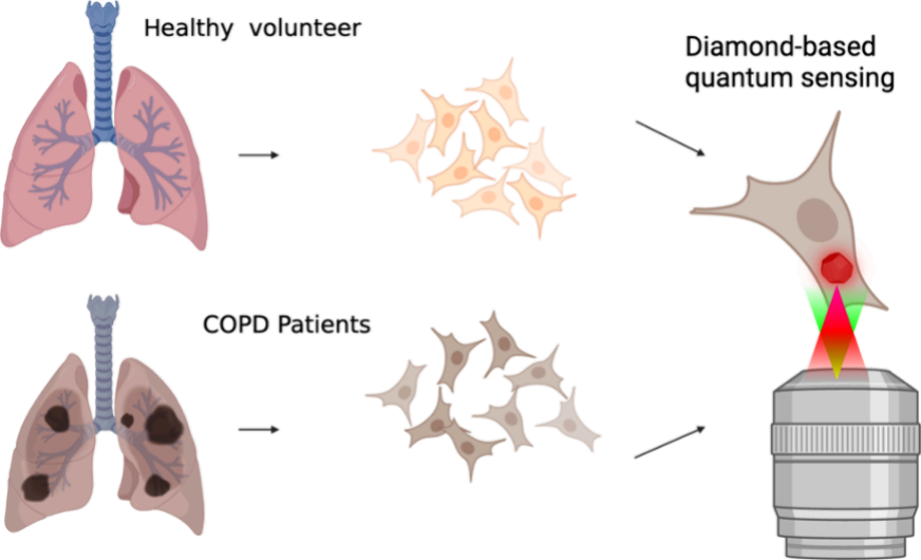

Chronic obstructive pulmonary disease (COPD), the third leading cause of death worldwide, is caused by chronic exposure to toxic particles and gases, such as cigarette smoke. Free radicals, which are produced during a stress response to toxic particles, play a crucial role in disease progression. Measuring these radicals is difficult since the complex mixture of chemicals within cigarette smoke interferes with radical detection. We used a new quantum sensing technique called relaxometry to measure free radicals with nanoscale resolution on cells from COPD patients and healthy controls exposed to cigarette smoke extract (CSE) or control medium. Epithelial cells from COPD patients display a higher free radical load than those from healthy donors and are more vulnerable to CSE. We show that epithelial cells of COPD patients are more susceptible to the damaging effects of cigarette smoke, leading to increased release of free radicals.

Chronic obstructive pulmonary disease (COPD) is currently one of the most severe health concerns, being the third leading cause of death worldwide. Patients who suffer from COPD usually experience progressive airflow limitation associated with an abnormal inflammatory reaction and impaired tissue repair mechanisms. This pathological progression results in mucus hypersecretion (chronic bronchitis), thickening of the small airway walls, and destruction of the alveolar tissue (emphysema) due to long-term exposure to noxious particles or gases, notably cigarette smoke and air pollution.^1^

In combination with genetic susceptibility, the inhalation of cigarette smoke and of other noxious particles is the main risk factor for COPD. Cigarette smoke contains 10^14^ free radicals/puff and causes oxidative damage, inflammation, and apoptosis, which drive the pathogenesis of COPD.^2,3^ Inhaled toxicants are first in contact with the airway epithelial barrier, inducing oxidative stress and mitochondrial dysfunction. These processes lead to pro-inflammatory responses and cellular damage,^4,5^ critical processes in the onset of COPD. However, the relationship between the cellular responses and the production of free radicals is complex and less explored.

Currently, free radicals can be detected by direct and indirect methods. Indirect methods usually measure the damage that radicals caused, such as lipid peroxidation or DNA damage.^6,7^ However, it is not always clear how the damage occurred or where the reactive molecules originated from. Another approach is to measure cellular responses, such as the upregulation of certain genes or the expression of certain proteins of the stress response.^8^ These methods are usually destructive and do not offer spatial resolution.

Additionally, free radicals can be measured using fluorescent- or spin-labels which react with a radical to form a fluorescent product or a stable radical. These adducts can then be detected with a fluorescence microscope^9^ or a magnetic resonance technique.^10^ Apart from that, the labels are often used in relatively high concentrations which might be toxic or suffer from bleaching and auto-oxidation issues.^11,12^ Furthermore, these labels reveal the cumulative effect rather than the current condition of the sample.

A novel method is based on nitrogen vacancy (NV) centers in diamonds which change their optical properties based on their surroundings.^13^ Since optical signals can be read out more sensitively this technique can detect even the small signal of single spins.^14−16^ There are several applications where this method has been utilized in physics, including magnetic measurements under extreme pressures^17,18^ or measurements of magnetic domains^19^ or nanostructures.^20^ Also for a few applications in biology diamond magnetometry has proven to be useful. Steinert et al. measured slices of fixed and spin labeled cells^21^ while Ermakova et al. detected iron containing proteins.^22^

Recently, we have shown that relaxometry, a specific type of diamond magnetometry measurements, can be utilized to measure free radicals inside living yeast cells, sperm cells, or immune cells.^23−26^

Here we have further optimized relaxometry and applied it for the first time to measure free radicals in the human bronchial epithelial cell line BEAS-2B and in primary airway epithelial cells obtained from COPD patients and healthy donors.

Diamond materials are described in the Supporting Information.

## Culture of BEAS-2B cells and human airway epithelial cells (HAECs)

The human bronchial epithelial cell line BEAS-2B was purchased from American Type Culture Collection (ATCC, Manassas, VA). Cells were cultured in RPMI-1640 medium (Lonza, Switzerland) supplemented with 10% fetal bovine serum (FBS, Gibco Life Technologies) and 1% penicillin/streptomycin (P/S, Gibco Life Technologies) at 37 °C with 5% CO_2_. The cells were passaged when the confluence reached 70%–90%.

Human primary airway epithelial cells (HAECs) were isolated by enzymatic treatment from tracheobronchial tissue of 5 transplanted patients with severe COPD and 5 control donors. The COPD patients (3 males and 2 females) are all severe GOLD stage IV COPD patients with a forced expiratory volume in 1 s (FEV_1_; % predicted) of 0.67 (±0.24) and a ratio of the FEV_1_ to the forced vital capacity (FEV_1_/FVC ratio, post-bronchodilation) of 45.33 (±10.12). The COPD patients had a mean age of 57 (±7.8) and are all ex-smokers with an average smoking history of 42.67 (±24.2) pack-years. No further information was available on the control donors. The study protocol was according to the research code of the University Medical Center Groningen (https://umcgresearch.org/w/research-code-umcg) and the national and ethical guidelines on the use of human body material (https://www.coreon.org/wp-content/uploads/2020/04/coreon-code-of-conduct-english.pdf).

HAECs were cultured in T25 culture flasks coated with 30 μg/mL fibronectin (Sigma-Aldrich, St. Louis, MO, USA), 30 μg/mL collagen (CellSystem, Troisdorf, Germany), and 10 μg/mL BSA (Sigma-Aldrich) in Airway Epithelial Cell Growth Medium (AEGM, PromoCell, Germany) with 1% penicillin/streptomycin (P/S, Gibco Life Technologies) at 37 °C with 5% CO_2_ and used for experiments at passage 3. Cells were seeded in coated 35 mm Petri dishes and grown to ∼90% confluency before being used. Experimental details for diamond uptake procedures, an MTT assay, *dsDNA& IL-8 ELISA* assays, and a cell titer test to assess biocompatibility as well as the 2′,7′-dichlorodihydrofluorescein diacetate (DCFDA) assay to determine ROS production are shown in the Supporting Information.

## Cigarette Smoke Extract (CSE)

CSE was freshly prepared as reported previously.^27,28^ In short, filters were removed from two 3R4F Kentucky research reference cigarettes, before being bubbled through a 25 mL RPMI-1640 serum free medium using a Watson Marlow 603S smoking pump at a rate of 8 L/h (Watson-Marlow, Delden, The Netherlands). The 100% CSE solution was diluted with serum free medium to prepare different concentrations of CSE.

## T1

Similarly as before,^29^ T1 measurements were performed on our homemade magnetometer (the equipment is available from QTsense, Groningen, The Netherlands, and described in the Supporting Information).

Once the FND of interest was identified, the relaxation measurement was performed.

We first performed T1 measurements in a cell-free environment. The stock solution of 70 nm FNDs was diluted in the cell culture medium and placed in a Petri dish. Then we replaced part of the solution with either fresh medium or CSE to reach the final concentration of 35%. T1 curves were recorded for 20 min after the intervention.

In the experiments with BEAS-2B cells, the cells were seeded in glass bottom Petri dishes at least 24 h before the experiment. On the day of the experiment, the cell culture medium was replaced with 5 μg mL^–1^ of 70 nm FNDs in the complete medium. The cells were incubated with FNDs for 2 h and washed with fresh medium. Using confocal microscopy, we found an FND internalized by one of the cells. Then part of the medium was replaced with either fresh medium or CSE to reach the final concentration of 5%, 10%, 15%, 20%, 35%, or 40%. The corresponding T1 curves were recorded for 10 min after the intervention, using one cell per sample to match the timing of the cellular response between the experiments. We repeated these experiments in primary HAECs, using the CSE concentrations of 5%, 15%, or 35%, and assessing the T1 changes for 20 min after the intervention. The statistical evaluation is described in the Supporting Information.

## Human Bronchial Epithelial Cells Internalize FNDs

It is widely accepted that cells exposed to external stimuli will experience oxidative stress.^30^ Detection of highly reactive and short-lived free radicals can aid early diagnosis, disease prevention, treatment, and mechanistic research. In this study, we developed and modified FND relaxometry to detect the dynamics of free radicals in single epithelial cells right after the stress stimulus, i.e., cigarette smoke extract, was applied.

**Figure 1:**
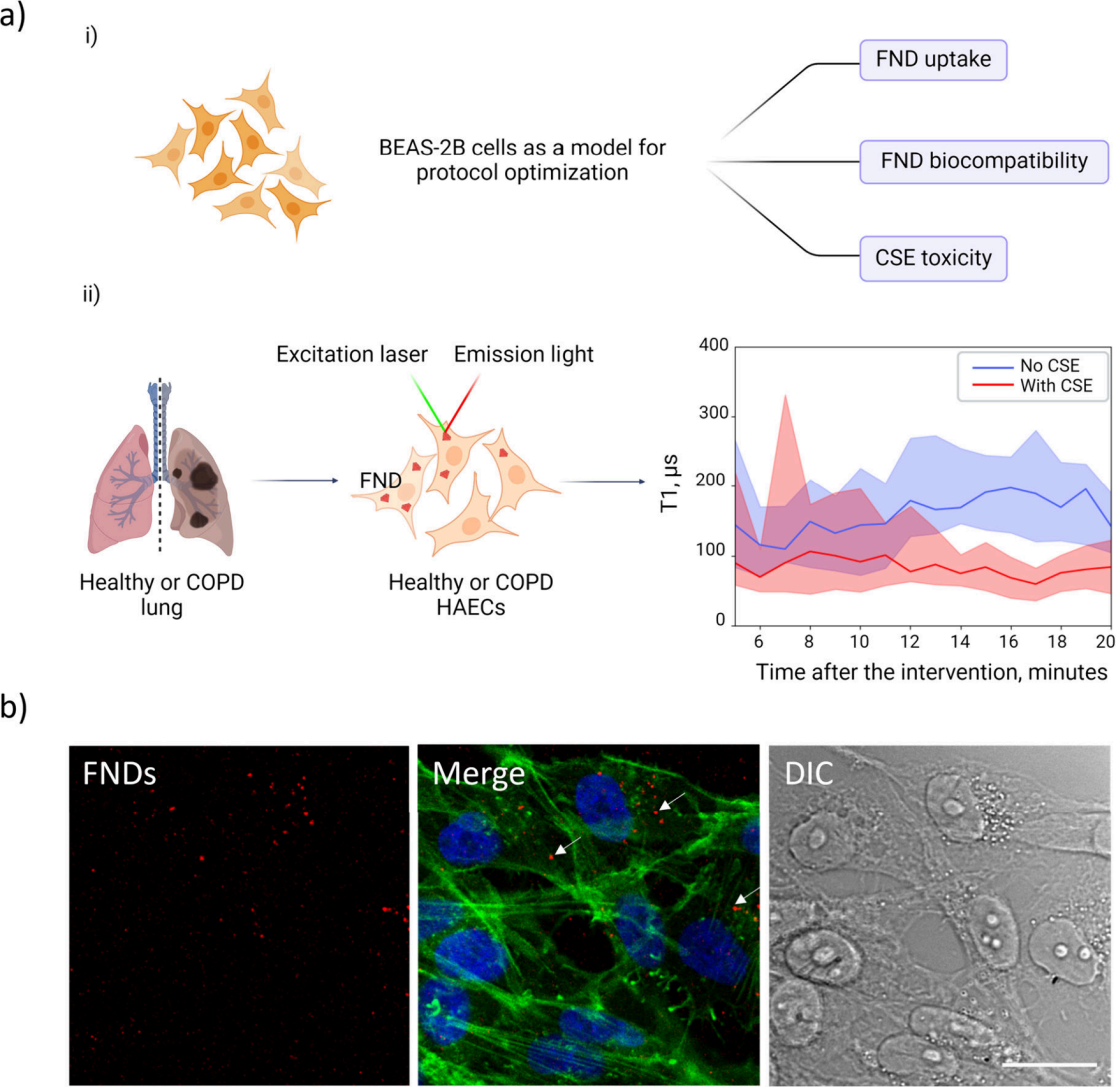
Dynamic quantum sensing of cigarette smoke extract (CSE)-induced free radicals in human primary airway epithelial cells (HAECs). (a) Quantum sensing of CSE-induced free radical generation in airway epithelial cells. (i) BEAS-2B cells were used as a model for primary human bronchial epithelial cells to assess FND uptake and toxicity and CSE toxicity. (ii) Human primary airway epithelial cells (HAECs) were isolated from tracheobronchial tissue of healthy control or COPD donors and then incubated with FNDs. The evolution of free radical load right after the cells were exposed to CSE was evaluated with relaxometry. (b) Confocal images of FNDs in BEAS-2B cells. Cells were incubated with 5 μg mL^–1^ FNDs for 2 h and then rinsed twice with PBS. Red: FNDs. Green: FITC-phalloidin labeled cytoskeleton. Blue: DAPI labeled nuclei. Gray: DIC images. White arrows indicate FNDs. The scale bar is 20 μm.

Human bronchial epithelial BEAS-2B cells were used as a model to optimize the procedure as these cells are sensitive to CSE and are commonly used to study stress responses and oxidative stress in the lung.^31,32^ Our experimental approach is depicted in Figure 1a.

In our study, we found that BEAS-2B cells can readily internalize FNDs. As shown in Figure 1b, FND fluorescence was clearly visible in the BEAS-2B cells after 2 h of incubation. In other types of cells such as HT-29 cells or HeLa cells, it usually takes longer incubation time or higher FND concentrations to complete endocytosis.^33,34^

## CSE Affects the Metabolic Activity and Free Radical Production in BEAS-2B Cells

**Figure 2:**
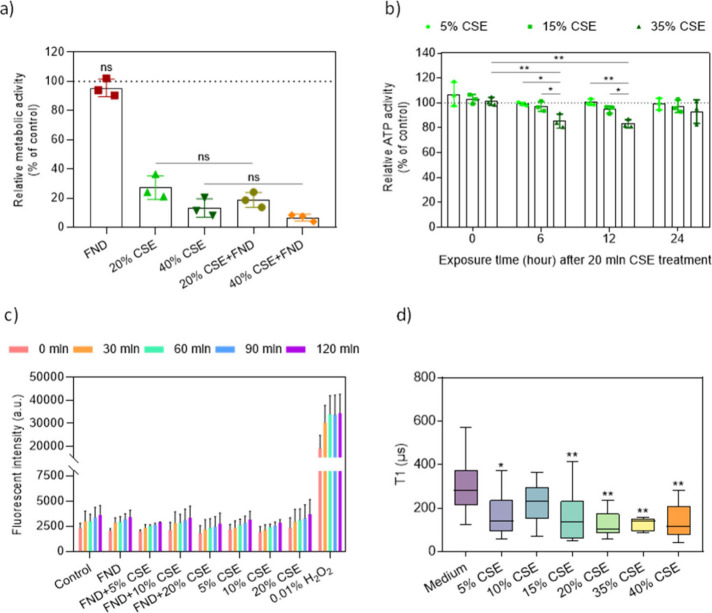
Evaluation of the cellular response to different concentrations of CSE in BEAS-2B cells. (a) The metabolic activity of cells exposed to FNDs and CSE was measured with an MTT assay. The cells were grown to confluency, serum-deprived overnight, and incubated with FNDs for 2 h and CSE for 4 h followed by another 24 h incubation. The data is shown as mean ± standard deviations (*t* test, ns >0.05, *p < 0.05, **p < 0.01). (b) The CellTiter assay was used to assess ATP changes within 24 h. The cells were exposed to CSE for 20 min and further incubated in the complete CSE-free medium for different times. The data is shown as mean ± standard deviations (two-way ANOVA, *p < 0.05, **p < 0.01). (c) A DCFDA assay was performed to evaluate the dynamics of ROS generation within 2 h after the cells were exposed to different CSE concentrations. Cells treated with 0.01% H_2_O_2_ were used as the positive control. (d) T1 measurements of the cells treated with different concentrations of CSE for 10 min. The data is shown as median ± interquartile range, and the whiskers show the minimum and maximum of the distribution (one-way ANOVA, *p < 0.05, **p < 0.01).

After the successful intracellular delivery of FNDs, we investigated whether FNDs will trigger a stress response. In general, FNDs are biocompatible and have been widely tested in many cells.^35−37^ As expected, in Figure 2a, the MTT assay showed that FNDs did not exhibit significant metabolic toxicity to the cells, while CSE was toxic at higher concentrations. After 4 h of exposure to 20–40% CSE, followed by extra 18 h of incubation in CSE-free medium, BEAS-2B cells showed a substantial reduction in the cellular viability. Besides, the presence of FNDs did not result in significant additional adverse effects in the CSE groups, indicating that FNDs are suitable for the detection of stress responses in human bronchial epithelial cells. When exposed to a high dose of CSE (>40%), the cells started to change morphology with shrinking and detachment within the first 2 h (Figure S1). We were particularly interested in the early stress response to CSE in viable cells, which is why we selected relatively low concentrations of CSE (<40%) for the following experiments.

To further study the early cellular responses to CSE, we performed a CellTiter assay to monitor the dynamics of cell viability over 24 h after a 20 min CSE treatment. This method evaluates the amount of ATP production, based on the luciferase reaction. As shown in Figure 2b, there was no noticeable cellular toxicity in the groups with low concentrations of CSE, e.g., 5% and 15%, meaning that the detection limit was reached. When 35% CSE was applied, the metabolic activity decreased notably during the first 12 h and then returned to the initial levels after 24 h. The dynamics suggest that the cells were under oxidative stress, the consequences of which could be detected for at least 12 h, but the cells eventually managed to recover.

Many studies have shown that smoking can induce oxidative stress, mitochondrial dysfunction, and cellular stress.^27,38,39^ Specifically, some constituents of CSE such as aldehydes^40^ and lipid-soluble components^4^ play critical roles in airway inflammation and the production of ROS. To investigate the oxidative stress response induced by cigarette smoke, we first conducted a commonly used DCFDA assay. This assay detects the fluorescence of the oxidized product of the reaction between the nonfluorescent probe and ROS inside a cell. PBS was selected as vehicle because it did not introduce substantial autofluorescence, unlike the components of cell culture media (Figure S2a). Cigarette smoke is a concentrated aerosol composed of thousands of toxic chemicals.^41^ Those compounds can increase the fluorescent background during the measurement, as illustrated in Figure S2b. After background subtraction, we could not detect an increase in ROS production by the cells in the different CSE groups as displayed in Figure 2c. The groups with FNDs also showed no additional induction of ROS. Surprisingly, even if the CSE concentration was increased to 100%, no significant response was found after 2 h of incubation (Figure S2c). We suspect that CSE reacts with DCFDA or the CSE itself interferes with the fluorescent readout. Another possible reason is that the autofluorescence of CSE is too high and eliminates the potential for measuring any additional fluorescence.

In relaxometry there is less interference from the fluorescent background because relaxometry measurements are done over a longer time and the background is bleaching. Also, T1 measures a relative relaxation rather than absolute fluorescent counts and is thus less sensitive to influences from the background. Hence, we assessed the production of free radicals after the BEAS-2B cells were exposed to a series of concentrations of CSE, ranging from 5% to 40%. For the first time, early changes of free radicals were observed. After 10 min of exposure, most of the CSE exposed groups already showed a notable drop in T1 values, suggesting a quick increase in free radical generation (Figure 2d). However, there was no clear dose dependence between the groups. There can be several possible reasons for this lack of dose dependency. First, the relationship between free radical concentration and T1 is not linear. Additionally, the random location of FNDs in the cells and cell heterogeneity may also bring some uncertainty. Finally, relaxometry reports on the immediate concentration of free radicals around the particle, while other methods reflect the cumulative history of the oxidative stress in the sample.

We further investigated the potential influence of CSE itself on the results of diamond relaxometry measurements. T1 measurements were conducted in the medium without cells and no obvious changes were detected after adding 35% CSE (Figure S3). Many people successfully captured free radicals in cigarette smoke with spin-trapping ESR.^3,42,43^ However, we did not observe free radicals in CSE .

## CSE Induces Oxidative Stress in Primary HAECs

**Figure 3:**
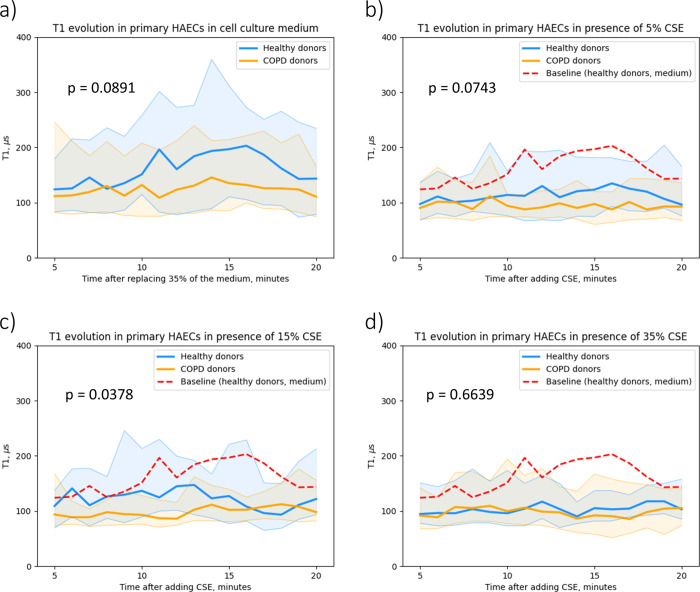
Comparison of T1 evolution between epithelial cells derived from healthy and COPD donors. Human airway epithelial cells (HAECs) were derived from tracheobronchial tissue of 5 healthy donors and 5 COPD patients. Cells were grown to confluence and exposed to (a) medium, (b) 5% CSE, (c) 15% CSE, and (d) 35% CSE for 20 min. The blue curve represents the median T1 values of the healthy donor group, whereas the orange curve denotes the median T1 values of the COPD donor group. The corresponding shadowed area indicates the interquartile range. The red dashed lines in (b)–(d) indicate the baseline from the healthy donor group for the better comparison. P values between healthy and COPD donors are indicated in each graph (two-way ANOVA, *p < 0.05).

Next, we performed experiments on primary human airway epithelial cells (HAECs) with the same concentration of FNDs. Similar to BEAS-2B cells, both healthy control and COPD-derived HAECs were able to take up FNDs after 2 h of incubation (Figure S4a). To assess cellular responsiveness and viability, the supernatants of HAECs were collected to evaluate the levels of IL-8 and dsDNA. In short, IL-8 is a marker of the inflammatory state,^44^ and dsDNA^45^ is released during necrotic type III cell death and can as such be used as a marker of cell death. As shown in Figure S4b, we found that epithelial cells from COPD donors were more sensitive to FNDs, since there were significant differences in the groups with or without FNDs while healthy HAECs did not show any response. The difference in response may derive from the disease having caused cellular impairment or genetic differences in susceptibility for radical production upon smoke exposure. Considering the possible effects of FNDs on the free radical production in primary cells, we monitored T1 curves in the control groups (healthy and COPD HAECs exposed to the cell culture medium, Figure 3a) and did not observe any notable differences. We concluded that there is no influence during the relatively short time the T1 measurement takes. Therefore, the free radicals released into the supernatant collected from a group of cells after 24 h of incubation can magnify the cellular response to the FNDs. In this situation, the short-term effects of FNDs in the single primary cells can be neglected.

Based on the T1 results in BEAS-2B cells, we chose three different CSE concentrations as low (5%), medium (15%), and high (35%) for HAECs. To better understand the evolution of free radicals and explore the responses, the T1 values were recorded for 20 min upon CSE treatment in both healthy and COPD donors. The data set was then analyzed with the rolling window approach, which allowed us to obtain the averaged T1 values for the specific incubation windows (0–5 min, 1–6 min, 2–7 min, etc.). Five COPD patients and five healthy controls were used.

Overall, as shown in Figure 3, the T1 curve of the healthy control group was generally above the T1 curve of the COPD group, both under unexposed and CSE-exposed conditions, indicating higher T1 values and lower free radical load in the healthy HAECs compared to COPD-derived HAECs during the treatment. Specifically, when exposed to 5% CSE, COPD-derived cells showed a trend toward shorter T1s than healthy subjects, but without reaching statistical significance (Figure 3b). The difference between the healthy control and the COPD group was further expanded and achieved significant difference with 15% CSE stimulation (p = 0.0378, Figure 3c). The only exception is in Figure 3d), as both groups responded to the highest concentration of CSE with an increased free radical load with narrow distribution, indicating that this intense external stimulus overwhelms the antioxidant defense of the cells, regardless of the health status of the donor. All stressed HAECs had T1 values around 100 μs which were similar to what we found in BEAS-2B cells.

**Figure 4:**
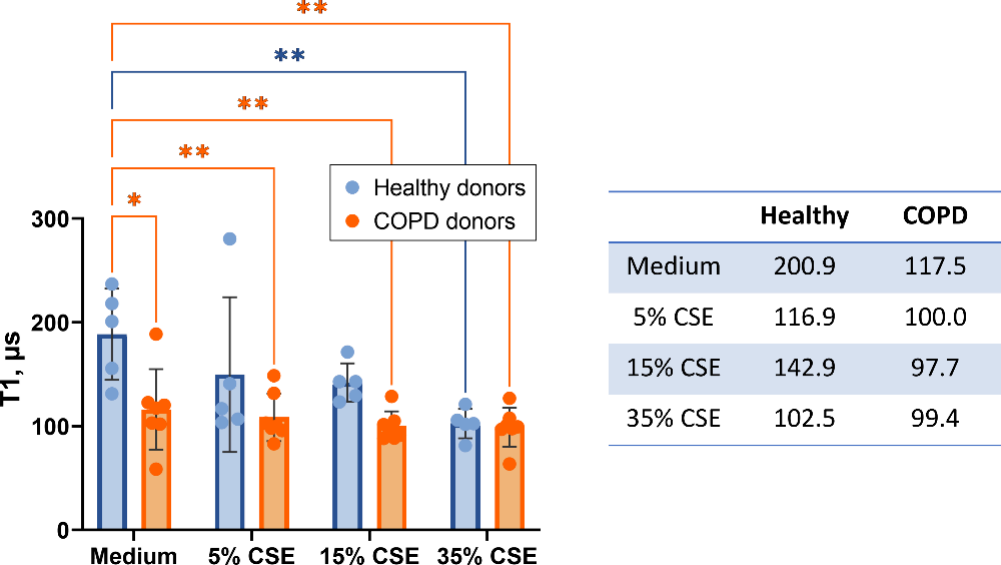
Comparison of T1 values integrated over the 20 min of incubation with different CSE concentrations between the healthy and COPD donors. On the left, each data point represents the pooled results of 6 to 8 measurements from one individual donor. Each bar is shown as median ± interquartile range (two-way ANOVA, *p < 0.05, **p < 0.01). On the right, the table shows the median of each group.

During the 20 min, fluctuations of T1 were observed but without a clear trend. T1 data collected over 20 min were integrated to assess the differences between different interventions and individuals. In Figure 4, we show that donors from healthy and COPD groups have different baseline levels of free radicals. Healthy HAECs are more sensitive to intervention and show a CSE dose dependence on T1 values. The table in Figure 4 shows that after an intervention of 35% CSE, the T1 value decreased by half. Meanwhile, the CSE effect was less pronounced in HAECs derived from COPD patients, which already showed lower T1 levels at baseline. This indicates that the baseline free radical level is already almost saturated in COPD-derived cells. At larger concentrations of CSE we do not see any significant differences between the COPD and control groups which is a limitation of our study. It is worth noting that the heterogeneity in the T1 levels may be explained by the use of primary cells with individual differences such as health status, sex, age, different genetic background, and various other factors. We observe similar response to the highest CSE concentration in the cells from healthy and COPD donors. It is worth noting that this concentration is also lethal to all cells, meaning that the oxidative stress is too high for any cell to adapt to. The observed free radical levels are affected by both the amount of free radicals produced in the cell and the cell’s antioxidant capacity (the amount of free radicals scavenged or neutralized by the cell). We hypothesize that cells from COPD donors have lower antioxidant capacity. In that case, the low degree of stress is sufficient to cause noticeable increase in free radical levels. However, under high-stress conditions, the antioxidant systems of both healthy and COPD cells will be overwhelmed, resulting in a similar response. We expect that by conducting a larger study, we might be able to reveal differences in the intermediate concentrations as well.

To the best of our knowledge, this is the first study to reveal the relationship between the total free radical load dynamics, the health status of the subjects. In summary, we show that CSE exposure leads to the generation of free radicals in airway epithelial cells. COPD-derived HAECs have a higher basic level of free radicals which could be a consequence of long-term cigarette smoking in combination with failing antioxidant responses.

The dynamic of free radical load is a rapid response of cells to external stimulation. Distinct from the conventional methods, diamond relaxometry can monitor the evolution of free radicals after the intervention and is not interfered with by the stressors. In this work, we successfully measured the cellular response to CSE on radicals in both BEAS-2B cells and primary human epithelial cells in 20 min. Overall, we show that CSE exposure leads to free radical production in human airway epithelial cells. Compared with healthy HAECs, COPD-derived HAECs have a relatively higher free radical level and both in the absence and presence of CSE. Our study demonstrated the feasibility of monitoring free radical dynamics upon CSE treatment in primary human epithelial cells. We believe that diamond relaxometry can provide more information on radicals and bring new insights into diagnostics, disease development, and future treatment strategies.

## Supporting Information Available

Experimental details; cell morphology measurements; conventional ROS measurements; control T1 measurements without cells; confocal images of HAEC from healthy donors and COPD patients; and IL-8 and double stranded DNA (dsDNA) (PDF)
