# Supplementary material for: Dynamics for High-Sensitivity Detection of Free Radicals in Primary Bronchial Epithelial Cells upon Stimulation with Cigarette Smoke Extract
Source: Nano Lett. 2024 Jul 16;24(31):9650–7. doi: 10.1021/acs.nanolett.4c02409 (PMC11311533; doi:10.1021/acs.nanolett.4c02409)
Supplement: Supplementary file 1 — nl4c02409_si_001.pdf [file nl4c02409_si_001.pdf]

Supplementary Information

Dynamics for high-sensitivity detection of free radicals in primary bronchial epithelial cells upon stimulation with cigarette smoke extract

Y. Zhang[#1], A. Sigaeva[#1], S. Fan[1], N. Norouzi[1], X. Zheng[2,3], I.H. Heijink [2,3,4], D.J. Slebos [3,4], S.D. Pouwels [2,3,4], R. Schirhagl[1*]

1. University of Groningen, University Medical Center Groningen, Department of Biomaterials and Biotechnology, Antonius Deusinglaan 1, 9713AV Groningen, the Netherlands
2. University of Groningen, University Medical Center Groningen, Department of Pathology and Medical Biology, Hanzeplein 1, 9713 GZ Groningen, The Netherlands

3. University of Groningen, University Medical Center Groningen, Department of Pulmonology, Hanzeplein 1, 9713 GZ Groningen, The Netherlands

4. University of Groningen, University Medical Center Groningen, Groningen Research Institute for Asthma and COPD (GRIAC), Hanzeplein 1, 9713 GZ Groningen, The Netherlands

#These two authors contributed equally

*Corresponding author email: romana.schirhagl@gmail.com

Fluorescent nanodiamonds

Carboxylated 70 nm fluorescent nanodiamonds (FNDs) were purchased from Adamas Nanotechnology. FNDs were produced by a high-pressure, high-temperature process. This type of diamond naturally contains nitrogen. Then particles were size separated and irradiated with 3MeV electrons at a fluence of $5*10^{19}$ e/cm^2 by the manufacturer. During this process vacancies were created. Next, the particles were annealed above 600 °C[1]. The resulting particles contain around 3ppm of nitrogen-vacancy centers (around 500 NV centers per particle). The last step of the particle synthesis by the manufacturer is cleaning in oxidizing acid resulting in oxygen terminated particles. The size, shape and surface chemistry have been extensively characterized before[2, 3].

Biocompatibility of FNDs

A 3-(4,5-dimethylthiazol-2-yl)-2,5-diphenyltetrazolium bromide (MTT) assay was performed to evaluate the metabolic activity of the BEAS-2B cells treated with different concentrations of CSE in combination

with or without FNDs. 30,000 cells per well were seeded in a clear flat-bottom 96-well plate and incubated at 37 °C with 5% CO_2 for 24h until ~90% confluency was reached. Afterwards, cells were incubated with FNDs for 2 h before they were exposed to CSE for 4 h. After another 18 h of incubation, the MTT solution (dissolved in PBS) was added to each well with a final concentration of 0.75 μg mL^{-1}. Afterwards, the plate was incubated at 37 °C for 3 h. Finally, the purple formazan was dissolved in isopropanol. The absorbance was measured with a synergy H1 microplate reader (BioTek) at OD = 590 nm. Untreated cells were used as the control.

2',7'-dichlorodihydrofluorescein diacetate (DCFDA)

DCFDA was used as an indicator for overall reactive oxygen species (ROS) within the cells. In general, BEAS-2B cells were incubated with FNDs for 2 h, followed by the treatment of 20 μM DCFDA in the complete medium for 45 minutes at 37 °C. After the incubation, DCFDA diffused into the cells and free reagent was removed by a rinse with PBS. Then, various concentrations of CSE or H_2O_2 were added to different wells and the fluorescence of DCF was measured with a synergy H1 microplate reader (BioTek) at 485nm excitation/535nm emission every 30 min for 2 h. Untreated cells were used as the control. Cells treated with 0.01% H_2O_2 were treated as the positive control.

CellTiter

A CellTiter assay measures the ATP concentration in the cells which is directly proportional to the number of viable cells in culture. Therefore, as a reference to T1, the CellTiter assay was used to evaluate the metabolic activity after the cells were exposed to the CSE. Similarly, BEAS-2B cells were incubated with FNDs for 2 h and then treated with different concentrations of CSE for 20 min. Next, the cells were rinsed twice with PBS and further incubated in the complete medium for 0 h, 6 h, 12 h and 24 h. Then, CellTiter was added per the protocol from the supplier (Promega) and luminescence was obtained with a microplate reader according to the protocol.

dsDNA & IL-8 ELISA

IL-8 was measured using the Human IL-8/CXCL8 DuoSet ELISA (DY208, R&D systems, Minneapolis, MN) according to manufacturer's protocol. Double stranded DNA was measured using the Quant-iT™ dsDNA Assay Kit (Q33120, Invitrogen, Waltham, MA) according to manufacturer's protocol. HAECs were cultured in quartered Petri dishes. When 70-90% confluency was reached, the cells were incubated with

FNDs for 2h followed with 20 min treatment of different CSE concentrations. After that, the cells were further incubated in AGEM for 24 h and the supernatants were collected, centrifuged for 5 minutes at 1000g to remove cells and stored at -80 °C until further usage. The supernatant of untreated cells was used as control.

Cellular uptake of FNDs

BEAS-2B cells were seeded at a density of 100,000 cells on quartered 35mm four compartment glass-bottom Petri dishes (Greiner) and cultured in an incubator overnight at 37°C, 5% CO_2. After the confluence reached ~90%, cells were serum deprived overnight, exposed to CSE for 4 hours and treated with 5 µg mL^{-1} FNDs for 2 hours, rinsed twice with PBS and then fixed with 3.7% PFA. Cells cultured in complete RPMI-1640 medium without FNDs were regarded as the control group. Fixed cells were further stained with FITC-phalloidin and DAPI to indicate the cytoskeleton and the nucleus, separately.

The fluorescent images were acquired using a Zeiss LSM780 confocal microscope with a 63× glycerin objective. FND signals were obtained using a 561 nm excitation laser and 670-740 nm as detection window. The FITC signals were obtained using a 488 nm excitation laser and a 500-550 nm detection window. The nucleus signals were obtained using a 405 nm excitation laser and 420-480 nm as detection window. The confocal images were deconvolved by FIJI plugins "Diffraction PSF 3D" and "Iterative Deconvolve 3D" to achieve a better contrast.

The FND uptake experiment in HAECs were conducted in the same way.

Description of the magnetometer that is used for relaxometry

The magnetometer is in principle a confocal microscope with a few adaptations to allow pulsing and tracking. A green laser (Torus 532 nm, Laser Quantum) attenuated to 70 µW (measured at continuous illumination at the position of the sample) is focused on the sample by a microscope objective (Olympus UPLSAPO 100XO, Oil immersion, NA 1.40). The photoluminescence is collected by the same objective, filtered by a 550 nm longpass dichroic mirror and a 650 nm longpass dielectric filter and sent to a photon counting avalanche photodiode (Excelitas Technologies SPCM-AQRH) through a confocal pinhole. The live cells are imaged by scanning the sample with the laser beam. Using these settings, both cells and nanodiamonds are visible. The background outside the cells is 10 000 counts per second on

average, the autofluorescence of the cytoplasm reaches 60 000 counts per second, while a diamond particle is typically 0.5-10 million counts per second. Cell morphology can be simultaneously assessed using the live bright-field images. As the focused laser spot is visible with the bright-field camera, one can confirm that the FND is located within the cell boundaries.

A train of 5 µs pulses is generated by an Acousto-Optic Modulator (Gooch & Housego AOMO 3350-199) in a double-pass configuration, and is routed to the FNDs to both initialize and readout the NV center's spin. A delay (or dark-time τ) between the pulses is logarithmically swept from 0.2 µs to 1 ms, and the photoluminescence is collected after every dark time. Such a dark-time sweep is repeated up to a total of 200 000 times (with 10 000 repetitions approximately taking 1 minute) for the longest experiments. This is an optimisation of the pulsing sequence that was previously used which leads to a reduction of measurement time by a factor of 10[4]. The photoluminescence of 50 000 sweeps is summed to obtain the photoluminescent pulses. The photoluminescence signal PL(τ) after different dark times τ is then integrated over the first 1 µs of each pulse. The photoluminescence signal is plotted against the corresponding values of τ, and the resulting relaxation curve is fitted by a double exponential function for extracting T1. The procedure is then repeated by shifting the summation window by 10 000 sweeps to obtain the T1 vs time curves. A certain amount of FNDs in the cells is necessary for the relaxometry. Theoretically, as little as one FND in a single cell is sufficient for one T1 measurement. However, for practical reasons, it is better to aim for several FNDs per cell, which reduces the time needed to find a particle and increases the chances for every cell having at least one FND.

Statistical analysis

Statistical analysis was performed in GraphPad Prism 9.4. The results of the T1 measurements were analysed using either Kruskal-Wallis test with post-hoc Dunn's multiple comparisons test or two-way ANOVA, with CSE concentration and incubation time taken as the predictors. The results of rest experiments were analysed using t-test or two-way ANOVA with post-hoc Tukey's multiple comparisons test. Statistical significance of the differences is reported as follows: "ns" for $p > 0.05$, "*" for $p < 0.05$, "**" for $p < 0.01$, "***" for $p < 0.001$, "****" for $p < 0.0001$.

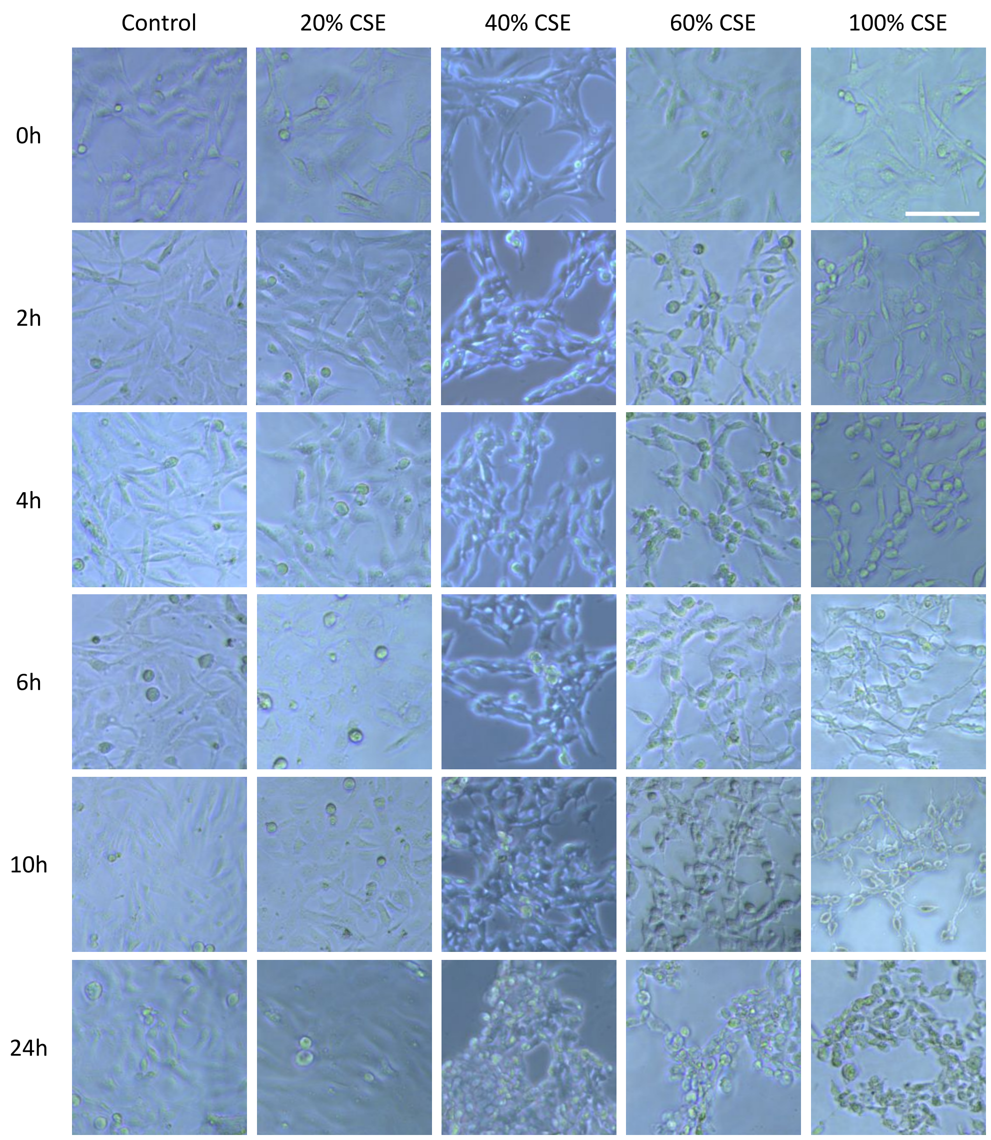

Figure S1. Cell morphology changes within 24 h after the cells were exposed to different concentration of CSE. The scale bar is 100 µm.

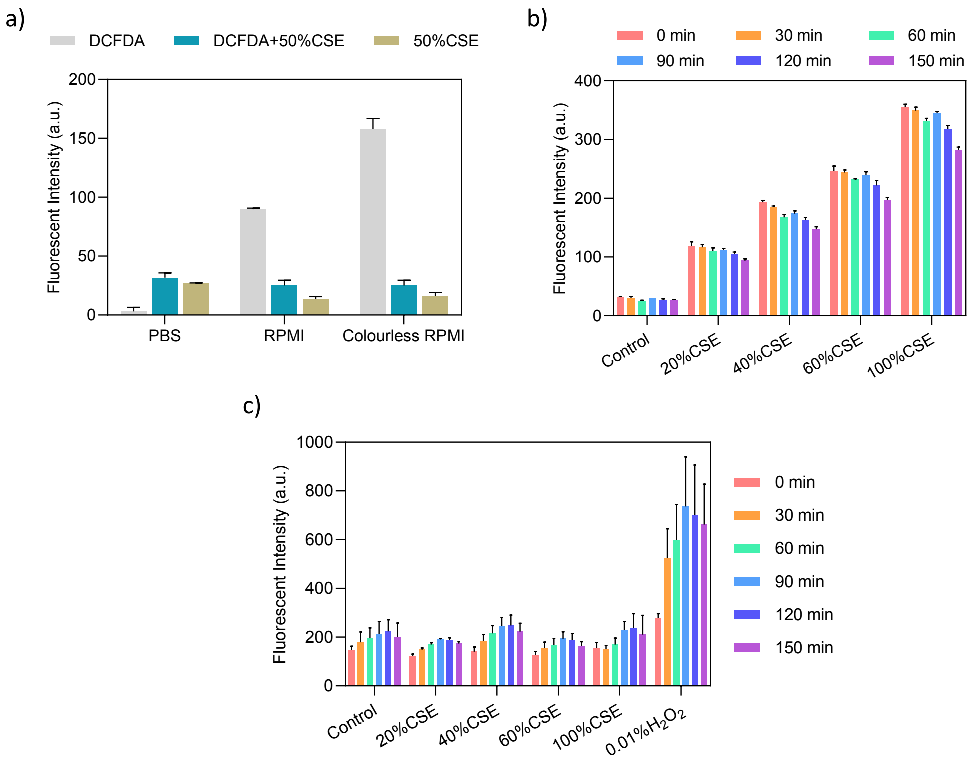

Figure S2. The DCFDA assays. a) medium screening. PBS, RPMI and colourless RPMI media were separately mixed with DCFDA and CSE. b) CSE caused fluorescent background within 2.5 h exposure. Higher concentration of CSE causes stronger background. c) Evaluation of ROS generation within 2.5 h after the BEAS-2B cells were exposed to 20%-100% CSE. 0.01% H_2O_2 were treated as the positive control.

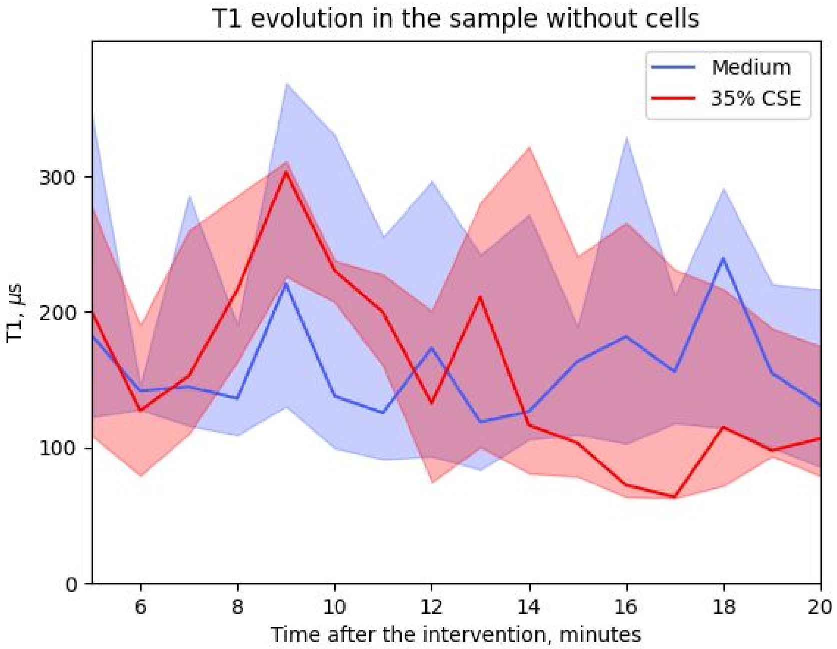

Figure S3. 20 min T1 evolution in the RPMI medium with or without 35% CSE. No cells were included. The data is shown as median ± interquartile range.

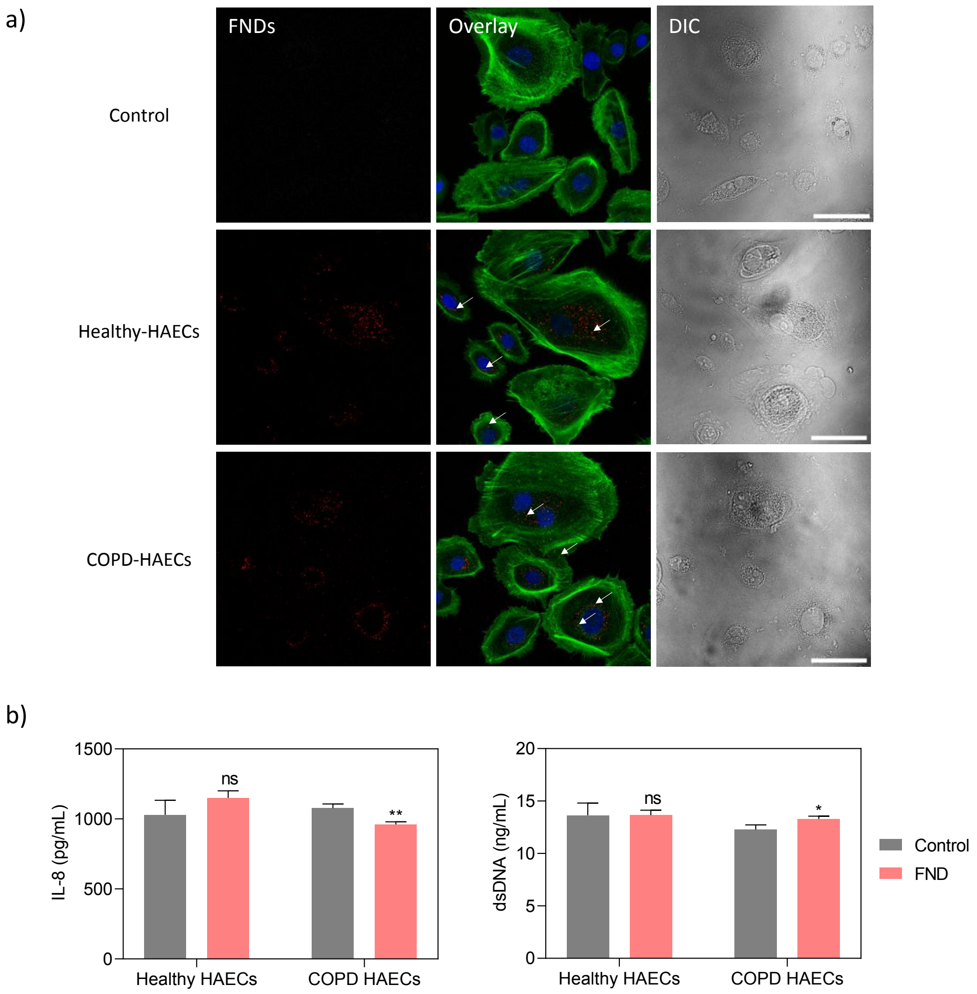

Figure S4. **a)** Confocal images of FNDs in healthy or COPD HAECs. The cells were incubated with 5 µg mL^{-1} FNDs for 2h and then rinsed twice with PBS. Red: FNDs. Green: FITC-phalloidin labeled cytoskeleton. Blue: DAPI labeled nucleus. Grey: DIC images. White arrows indicate FNDs. The scale bar is 50 µm. **b)** Evaluation of IL-8 and double stranded DNA (dsDNA) in the cell free supernatant. HAECs were incubated with or without FNDs for 2 h, and then further cultured for 24 h. The supernatant was collected for the following tests. The data is shown as mean ± standard deviations (t-test, ns > 0.05, *p < 0.05, **p < 0.01).

References

1. Shenderova, O.A., et al., *Review Article: Synthesis, properties, and applications of fluorescent diamond particles.* Journal of Vacuum Science & Technology B, 2019. **37**(3): 27.
2. Hemelaar, S.R., et al., *Nanodiamonds as multi-purpose labels for microscopy.* Scientific Reports, 2017. **7**: 9.
3. Ong, S.Y., et al., *Interaction of nanodiamonds with bacteria.* Nanoscale, 2018. **10**(36): 17117-17124.

4. Morita, A., et al., *Detecting the metabolism of individual yeast mutant strain cells when aged, stressed or treated with antioxidants with diamond magnetometry.* Nano Today, 2023. **48**: 13.
